# Environmental UVR Levels and Skin Pigmentation Gene Variants Associated with Folate and Homocysteine Levels in an Elderly Cohort

**DOI:** 10.3390/ijerph17051545

**Published:** 2020-02-28

**Authors:** Patrice Jones, Mark Lucock, Christopher J. Scarlett, Martin Veysey, Emma Beckett

**Affiliations:** 1 School of Environmental & Life Sciences, University of Newcastle, Ourimbah, NSW 2258, Australia; mark.lucock@newcastle.edu.au (M.L.); emma.beckett@newcastle.edu.au (E.B.); c.scarlett@newcastle.edu.au (C.J.S.); 2 Hunter Medical Research Institute, New Lambton Heights, NSW 2305, Australia; 3 Hull-York Medical School, University of Hull, Hull HU6 7RX, UK; martin.veysey@hyms.ac.uk

**Keywords:** folate, skin pigmentation, ultraviolet radiation, nutrigenetics, gene–nutrient–environment interactions

## Abstract

Ultraviolet radiation (UVR) is a ubiquitous exposure which may contribute to decreased folate levels. Skin pigmentation mediates the biological effect of UVR exposure, but its relationship to folate levels is unexamined. Interactions may exist between UVR and pigmentation genes in determining folate status, which may, in turn, impact homocysteine levels, a potential risk factor for multiple chronic diseases. Therefore, independent and interactive influences of environmental UVR and genetic variants related to skin pigmentation (*MC1R*-rs1805007, *IRF4*-rs12203592 and *HERC2*-rs12913832) on folate (red blood cell (RBC) and serum) and homocysteine levels were examined in an elderly Australian cohort (n = 599). Genotypes were assessed by RT/RFLP-PCR, and UVR exposures were assessed as the accumulated erythemal dose rate accumulated over 4 months (4M-EDR). Multivariate analysis found significant negative associations between 4M-EDR and RBC folate (*p* < 0.001, β = −0.19), serum folate (*p* = 0.045, β = −0.08) and homocysteine levels (*p* < 0.001, β = −0.28). Significant associations between *MC1R*-rs1805007 and serum folate levels (*p* = 0.020), and *IRF4*-rs12203592 and homocysteine levels (*p* = 0.026) occurred but did not remain significant following corrections with confounders. No interactions between 4M-EDR and pigmentation variants in predicting folate/homocysteine levels were found. UVR levels and skin pigmentation-related variants are potential determinants of folate and homocysteine status, although, associations are mixed and complex, with further studies warranted.

## 1. Introduction

Folate is an essential nutrient with a major role in regulating homocysteine levels, a nonessential amino acid which at elevated levels is an independent risk factor for adverse health outcomes such as atherosclerosis, stroke and coronary artery disease [1]. Folate acts as a key regulator of homocysteine levels through roles in converting homocysteine to essential amino acid, methionine, with an inverse relationship between folate and homocysteine levels well established [2,3]. Folate is also important in the synthesis of DNA nucleotides, with these collective roles linking low folate status to risk of additional adverse health outcomes, such as neurodegenerative disorders, adverse pregnancy outcomes and several cancer forms [1,4,5]. Various environmental and genetic factors determine folate status [6], with potential consequences for health and disease risks. Many of these factors have been studied in isolation, however, determinants may also interact to influence folate status and activity, leading to different outcomes depending on the combination of exposures. As such, environment–gene–nutrient interactions are an emerging area of research interest which may explain the diversity of outcomes reported and yield more personalised health recommendations in the future. 

Exposure to ultraviolet radiation (UVR) is a potential environmental determinant of folate status. Folate photodegradation may be of particular importance in Australians [7,8] due to high UVR exposures experienced by much of this population [9]. In vitro studies have demonstrated UVR exposure may directly degrade folates and stimulates the production of reactive oxygen species by endogenous photosensitisers, which may increase folate needs and losses [10,11,12,13]. These findings have been supported by several observational human studies, where increased UVR levels or exposures have been associated with decreased folate status [7,8,14,15]. Potential season variation in related compound, homocysteine, has been examined in two previous studies with no significant relationships reported [16,17]. However, these prior observational studies have had limited consideration of the potential influence of interacting determinants on the impact of UVR on folate status or subsequent homocysteine levels.

The biological influence of UVR exposure is dependent on several factors, with skin pigmentation the most important endogenous determinant of UV sensitivity. Variation in human skin pigmentation is an evolutionary adaptation to differing UVR environments, with darker-pigmented populations originating in areas of high environmental UVR, and lighter-pigmented in regions of lower UVR levels [18,19]. Debate still surrounds the selective values of skin pigmentation, although the prominent theory proposes skin pigmentation evolved as a balancing mechanism to maintain levels of two vitamins central to reproductive success; folate and vitamin D [18,19]. This theory proposes that darker pigmentation allowed for the protection of folate against high UVR loads near the equator, and depigmented/lighter-pigmented skin promoted adequate photosynthesis of vitamin D in low UVR environments nearer the poles [18,19]. 

Folate status has been shown to vary between subjects of differing ethnicities [20,21,22]. Analyses of >8000 subjects from the National Health and Nutrition Examination Surveys in the USA found both red blood cell (RBC) and serum folate concentrations were highest in non-Hispanic whites compared with non-Hispanic blacks and Mexican Americans [20,21]. Furthermore, Perry et al. [22] showed that ethnicity influences response to folate intake, with serum folate levels lower in African American women, compared with Mexican American and non-Hispanic whites, following folate depletion and repletion diets. While UVR exposure and ethnic background are each shown to influence folate levels, the direct role of genetic variants related to skin pigmentation and UVR sensitivity is yet to be assessed. Furthermore, the interactive effects of UVR and skin pigmentation in determining folate and homocysteine status have not been examined. 

Human skin pigmentation is a polygenic trait, influenced by multiple genes in many diverse pathways which regulate melanin production. Polymorphisms in major genes of these pathways cause apparent changes in skin pigmentation and display ethnic differences in frequency [23]. Among these genes, the melanocortin 1 receptor gene (*MC1R*) is the most extensively studied, involved in the synthesis of UVR-protective eumelanin (i.e., black/brown pigment), and is highly polymorphic in Europeans [24]. Polymorphisms in *OCA2/HERC* and *IRF4* have been linked to changes in constitutive skin pigmentation and the related processes of tanning response, photo-aging and skin cancer risk in several studies [25,26,27,28]. Common variants in these genes are therefore considered genetic markers of UVR skin sensitivity [23,24,25,26,27,28]. 

Therefore, we extend our previous examination of the relationship between environmental UVR levels and RBC folate levels in an elderly Australian cohort [7]. Here, we examine independent and interactive influences of skin pigmentation-related variants (*MC1R*-rs1805007, *IRF4*-rs12203592 and *HERC2*-rs12913832) and environmental UVR in determining folate (RBC folate and serum folate) as well as homocysteine levels. 

## 2. Materials and Methods

### 2.1. Subjects and Sample Collection

This study was a secondary analysis of samples and pre-existing data from the Retirement Health and Lifestyle Study (RHLS), a cross-sectional study examining the health and lifestyle of older Australians (>65 years) living in the Central Coast region of New South Wales, Australia [29,30,31]. This cohort is suitable for this analysis as Australians are exposed to high levels of UVR radiation [9], and aging populations are at a heightened risk for UVR damage due to UVR-protective mechanisms in the skin declining with age [32]. Subjects were eligible to be included in the RHLS if they lived independently within the community or resided in retirement villages on the Central Coast. Subjects were included in this secondary analysis if they provided blood for biochemical and genotypic analyses and provided complete and valid food frequency questionnaires (n = 599), with questionnaires deemed invalid based on excess/deficient reported intakes. Subjects provided written informed consent, and ethics approval for the study was obtained from the University of Newcastle Human Research Ethics Committee (reference No. H-2008-0431).

### 2.2. Sample Collection and Biochemical Measurements

Fasting blood samples were collected in ethylenediaminetetraacetic acid lined tubes (whole blood and serum) or heparin (plasma). Plasma and serum were isolated from whole blood by centrifugation. Whole blood samples were stored at −20 °C, and serum and plasma at −80 °C. Serum folate, creatinine and vitamin B_12_ and RBC folate levels were assessed by The Hunter Area Pathology Service via standardised protocols [7]. Total plasma homocysteine levels were measured by a single-enzyme-selective fluorescence assay and OP-162 homocysteine reader (JD Biotech Corp, Taipei, Taiwan) [33,34]. 

### 2.3. Genotyping of Skin Pigmentation Polymorphisms

Genomic DNA was isolated from whole blood samples using QIAamp mini-kits following manufacturer’s protocols (Qiagen). Isolated DNA was used to genotype for three genetic variants related to skin pigmentation; *IRF4* rs12203592, *HERC2* rs12913832 and *MC1R* rs1805007. These variants were selected due to their well-established associations with skin pigmentation and related traits of interest; tanning ability/tanning response to sun and photoaging [25,26,27,28].

*IRF4*-rs12203592 genotyping was performed with the Taqman genotyping assay (C_31918199_10; Applied Biosystems). PCR reactions of 5 µL were performed in 384-well plates and comprised ~20 ng of DNA (applied as 2.5 µL of DNA sample dried in plate), 2.50 µL of 2X TaqMan^®^ Master Mix, 0.25 µL of assay mix and 2.25 µL of Nuclease-free water as per manufacturer’s instructions. Reactions and genotype assignments were performed with a QuantStudio™ 7 Flex Real-Time PCR system (Applied Biosystems). *HERC2*-rs12913832 and *MC1R*-rs1805007 genotyping was carried out by RFLP-PCR analysis, following methods outlined in Table 1 and adapted from Iida et al. (2009) and Dȩbniak et al. (2006) [35,36]. PCR products were digested with *DraI* (*HERC2* rs12913832) and *HhaI* (*MC1R* rs1805007) for 16 h at 37 °C, and then separated on 4% agarose gels stained with ethidium bromide to determine genotypes. Determination of the *MC1R* rs1805007 TT genotype was not sought due to the reported low frequency of this genotype in global populations (<1%; dpSNP—https://www.ncbi.nlm.nih.gov/snp/rs1805007 (accessed on 1 November, 2019). 

### 2.4. Estimation of Subject Sun Exposure: Accumulated Erythemal Dose Rate

Coordinates of the subject’s reported location were used to estimate environmental UVR levels based on erythemal dose rate (EDR) in the geographic area prior to blood collection. EDR is a measure of the potential for biological damage due to UVR and is calculated using the levels of UV irradiation at different wavelengths, weighted by model values of the susceptibility of lightly pigmented skin to sunburn (i.e., erythema) [37]. EDR was used as a surrogate measure of participant UVR exposure, with data gathered for the total amount of EDR accumulated over the 4 month period prior to each subject’s blood sample collection (i.e., clinic date). A 4 month period was chosen as it equates to the approximate lifespan of a red blood cell, likely providing the strongest corollary with examined blood nutrient levels. Accumulated EDR values were calculated by summing the average daily erythemal dose rates (at noon) reported from NASA’s Total Ozone Mapping Spectrometer, accessed via NASA’s online Giovanni platform (https://giovanni.gsfc.nasa.gov/giovanni/) [29,30]. Derived values varied considerably in the RHLS cohort given original sample collection took place over a period of 20 months from July 2010 to March 2012 (>80 sample collection dates).

### 2.5. Questionnaires and Clinical Data

Dietary intake of subjects was estimated using a validated self-administered food frequency questionnaire (FFQ) covering 225 food items and all food groups [38]. Subjects indicated if they obtained additional intake of nutrients via supplement use, and this was converted to total dietary equivalents where appropriate. FFQs were analysed using Foodworks^TM^ software (V.2.10.146; Xyris Software, Brisbane, QLD, Aus).FFQs were deemed invalid if they were incomplete, reported an excess (>30,000 kJday) or deficient (<3500 kJ/day) energy consumption, or an excessive reported consumption of a single food group (≥11 serves/day) [38]. Specific dietary information used in these analyses was information on B vitamin intake (folate, vitamin B_6_), alcohol consumption and average daily serves of tea and coffee (1 serve = 250 mL), as these are known to potentially influence folate [39,40] and homocysteine levels [39,41,42]. 

Information on smoking status was collected via an interviewer-administered survey with classification as either non-smoker, ex-smoker or current smoker. Participant body mass index (BMI) (kg/m^2^) was calculated using anthropometric measurements undertaken at time of interview following standard procedures [31], with subjects further classified in BMI categories; underweight (BMI; <18.5), normal weight (BMI; 18.5–24.9), overweight (BMI; 25–29.9) or obese (BMI >30).

### 2.6. Statistics

Analyses were performed using JMP (V.14.2.0; SAS Institute Inc., Cary, NC, USA). Descriptive statistics (means and 95% confidence intervals) were calculated and presented as appropriate. Multifactorial modelling via standard least squares regression was used to assess the relationships between folate and homocysteine levels, and variables of interest. Where appropriate, interaction terms were included in models. Where folate levels were the outcome, analyses adjusted for age, sex and known determinants of folate status; dietary intake of folate, vitamin B_6_ and alcohol, tea and coffee consumption (serves/day), serum vitamin B_12_ levels, BMI category and smoking status [39,40]. Where homocysteine levels were the outcome, analyses adjusted for age, sex and known determinants of homocysteine status; reported intake vitamin B_6_ and alcohol, serum folate, vitamin B_12_ and creatinine levels, BMI category and smoking status [39,41,42]. Multiple comparisons of least squares means were made using Tukey’s HSD tests. Adjusted R^2^ values and *p*-values are reported for final models, with standardised parameter estimates (β) and *p*-values reported for individual variables. Outcomes were considered to be statistically significant at *p* ≤ 0.05.

## 3. Results

### 3.1. Cohort Characteristics

The cohort was 45% male with a mean age of 77 years (Table 2). Average RBC folate, serum folate and homocysteine levels were within the normal ranges, defined as 317–1422 nmol/L for RBC folate, 7–45 nmol/L for serum folate and 4–15 μmol/L for homocysteine levels [43]. There were a low number of current smokers in this cohort, therefore, current smokers or those who had a history of smoking were combined into one category for further analysis. Additionally, only 2% of subjects had a BMI classified as underweight, therefore these individuals were combined with those of normal BMIs in analyses. 

Blood creatinine levels, vitamin B_12_ levels, alcohol intake and smoking status varied by sex (Table 2). Mean creatinine levels and alcohol intake were significantly higher in males compared with females (10.5 vs. 8.4; *p* < 0.001 and 13.3 vs. 4.7; *p* < 0.001; Table 2). Serum vitamin B_12_ levels were lower in males (224.0 vs. 252.0; *p* = 0.025). Only 3% of the cohort identified as a current smoker, but a significantly higher percentage of males had a history of smoking compared with females (63% vs. 32%; *p* < 0.0001; Table 2). The distribution of other dietary and lifestyle variables of interest did not differ by sex (Table 2). 

The distributions of *IRF4*-rs12203592, *HERC2*-rs12913832 and *MC1R*-rs1805007 variants in the cohort are outlined in Table 3. Genotype frequencies were comparable to 1000 Genomes European (EUR) populations, particularly Northern Europeans (i.e., GBR; Table 3). Due to the low frequency of *IRF4*-rs12203592 TT and *HERC2*-rs12913832 AA genotypes (<10%), genotypic analyses excluded these genotypes and compared only the two major genotypic variants (i.e., *HERC2*-rs12913832 AG vs. GG and *IRF4*-rs12203592 CT vs. CC). 

### 3.2. Association between Accumulated Erythemal Dose Rate and Folate Levels

Erythemal dose rate accumulated over 4 months (4M-EDR) was negatively associated with RBC folate levels, with increases in 4M-EDR associated with reductions in RBC folate (*p* < 0.001, β = −0.19; Table 4). This association remained significant when adjusted for age, sex and known determinants of folate status. A significant negative association was also found between 4M-EDR and serum folate levels, with increases in 4M-EDR also associated with reductions in serum folate levels in unadjusted and adjusted models (Table 4). 

### 3.3. Association between Accumulated Erythemal Dose Rate and Homocysteine Levels

Increases in 4M-EDR were significantly associated with reductions in serum homocysteine levels (*p* < 0.001, β = −0.28; Table 5). This association remained significant following adjustments for age, sex and known determinants of homocysteine levels (*p* < 0.001, β = −0.28).

### 3.4. Association between Skin Pigmentation Variants and Folate Levels

No associations were found between skin pigmentation variants and RBC folate levels (Table 6). The *MC1R*-rs1805007 CC genotype was associated with significantly lower serum folate levels compared with the CT genotype (27.4 (26.2–28.6) vs. 31.0 (28.3–33.6); Table 6). *HERC2*-rs12913832 and *IRF4*-rs12203592 variants were not independent significant predictors of serum folate levels. The association between *MC1R* rs1805007 and serum folate levels did not remain following adjustments for age, sex and known determinants of folate status. 

### 3.5. Association between Skin Pigmentation Variants and Homocysteine Levels

The *IRF4*-rs12203592 CC genotype was associated with significantly lower homocysteine levels compared with the CT genotype (9.6 (8.8–10.5) vs. 10.9 (9.9–11.8); p = 0.026; Table 7). However, this significant association did not remain following adjustments for age, sex and known determinants of homocysteine levels. *MC1R*-rs1805007 and *HERC2*-rs12913832 variants were not independent significant predictors of homocysteine levels.

### 3.6. Interaction between Accumulated Erythemal Dose Rate and Skin Pigmentation Variants on Folate Levels

To assess if the effects of 4M-EDR and skin pigmentation variants on folate levels were independent or interactive, multivariable modelling with the inclusion of interactive terms for 4M-EDR and *MC1R*-rs1805007, *HERC2*-rs12913832 and *IRF4*-rs12203592 was performed. No significant interactions between 4M-EDR and variants *MC1R*-rs1805007, *HERC2*-rs12913832 and *IRF4*-rs12203592 in predicting changes in RBC folate levels were found, with only 4M-EDR remaining a significant independent predictor of folate levels in unadjusted and adjusted models (Table 8). For serum folate levels, no significant interactions between 4M-EDR and skin pigmentation variants were shown, with the main effect of *MC1R* rs1805007 genotype on serum folate levels remaining significant in unadjusted, but not adjusted, models (*p* = 0.024 and *p* = 0.155; Table 8). 

### 3.7. Interaction between Accumulated Erythemal Dose Rate and Skin Pigmentation Variants on Homocysteine Levels

No significant interactions between 4M-EDR and *MC1R*-rs1805007, *HERC2*-rs12913832 and *IRF4*-rs12203592 variants in predicting changes in homocysteine levels were shown. However, the main effect of 4M-EDR remained significantly associated with homocysteine levels in unadjusted and adjusted models (*p* < 0.001; Table 9). 

## 4. Discussion

This study is the first to examine both independent and interactive influences of environmental UVR and pigmentation-related genetic variants on folate and homocysteine levels. Environmental UVR levels and pigmentation-related variants *MC1R*-rs1805007 and *IRF4*-rs12203592 were shown to be independently associated with folate and homocysteine levels, however no interactive influences between these factors were found. 

Associations between UVR levels and folate status have previously been reported in multiple studies examining serum folate status only [8,14,15,44]. The data reported here support these previous studies with both serum and RBC folate levels examined. Serum folate is an indicator of short-term folate status, reflective of recent dietary intake, while RBC folate reflects long-term status as it is less influenced by recent dietary intake [45]. 4M-EDR was negatively associated with levels of both serum and RBC folate, indicating UVR as a potential environmental determinant of both short- and long-term folate status. Conversely, other studies report no association between UVR and folate levels [46,47,48]. However, as highlighted in a previous review by Zhang et al. [49], these studies typically examine cohorts with lower cumulative UVR doses compared with those where associations were reported. This hypothesis is supported by the associations reported in the present study, which assessed accumulated UVR levels over a long period (4 months) in a high UVR environment (i.e., Australia). 

This is the first report of UVR-associated variability in homocysteine levels. While the relationship between homocysteine levels and season have been investigated previously, no significant relationship has been reported [16,17]. Obvious differences between prior studies and the current study are cohort size and location. Prior studies examined <100 subjects located in Northern European areas [16,17]. Differences in findings may therefore reflect differences in power and environmental UVR levels. Furthermore, season is a non-precise surrogate for UVR levels compared with the satellite data used here.

Given the well-known inverse relationship between folate and homocysteine levels [2,3], the finding that both folate and homocysteine decreased with increased environmental UVR was unexpected. This suggests homocysteine may be decreased by processes which are upregulated following UVR exposure. Vitamin D, which is consistently inversely associated with homocysteine levels [50,51,52,53,54], may be involved in this process or may be a marker of sun-exposure, supporting this hypothesis. Increased oxidative stress following UVR exposure may also influence homocysteine levels, by promoting homocysteine auto-oxidization and the formation of homocysteine into another oxidant not detected by homocysteine assays [55]. Further investigation into the potential relationship between UVR and homocysteine levels is warranted, given homocysteine is an independent risk factor for several cardiovascular and neurovascular diseases [56]. 

To our knowledge, this is the first study to explore relationships between UVR, skin pigmentation gene variants and folate/homocysteine levels. Significant associations were found between *MC1R*-rs1805007 genotype and serum folate levels, and between *IRF4*-rs12203592 genotype and homocysteine levels. However, associations did not remain in adjusted models, indicating the influence of the variants was not greater than that of already known determinants of folate and homocysteine levels. It is unclear why the *MC1R*-rs1805007 CT genotype was related to higher serum folate levels compared with the CC genotype, given it is the T allele that is strongly related to the red hair/fair skin phenotype and increased sun sensitivity [23,25,26,28]. These findings may reflect differences in sun habits rather than a biological effect, where individuals with genetically fairer skin may have exercised cautionary sun habits. An unexpected relationship between the *IRF4*-rs12203592 CT genotype and increased homocysteine levels was also shown, with the T allele linked to increased sun sensitivity [26,27,28]. However, this may reflect an age-specific effect of *IRF4*-rs12203592 on sun sensitivity and related processes. The influence of the *IRF4*-rs12203592 T allele has been shown to be age-dependent in a large multi-population analysis of melanoma case-control data [57]. This study showed an association with high nevus counts in adolescents, but low nevus counts in adults. 

Age of subjects may partly explain why no interactions were found between environmental UVR and genetic variants. The potential UVR-modifying effect of skin pigmentation variants may be masked by the increased susceptibility of an elderly population to UVR damage, given aged skin displays a continuous loss of characteristics that allow for UV protection, and hyper or hypopigmentation conditions are common [32]. This may be particularly relevant to an Australian elderly cohort, given chronic high UVR exposures over the whole lifespan accelerates skin aging processes (i.e., photoaging) [32,58]. Further, findings of no interaction between UVR and genetic variants may be due to the large cumulative measure of UVR used, which may have masked any small mediating effects of genetic factors. 

Strengths of this study include the large cohort, which was well-characterized in respect to other known determinants of folate and homocysteine levels. Furthermore, a broad range of environmental UVR levels were examined, with 4M-EDR values varying considerably among subjects due to sample collection spanning 20 months. However, this study was limited in examining environmental UVR levels rather than personal UVR exposures. This was necessary due to this being a secondary retrospective cohort analysis, however, this also allowed the use of objective measures of UVR and for assessment of UVR levels over a long period (4 months) in a cohort of several hundred people. As skin pigmentation is a polygenic trait, multiple variants in numerous other related genes could be examined in future analyses [23]. Given this study was the first to examine relationships between folate/homocysteine and skin pigmentation variants, three genetic variants (*IRF4* rs12203592, *HERC2* rs12913832 and *MC1R* rs1805007) were selected on the basis of being consistently associated with pigmentation and sun sensitivity [25,26,27,28]. These variants have importance in determining skin pigmentation in populations predominately of European ancestry, such as in this cohort, and therefore, findings may be limited to these populations. Finally, as this study focused on assessing an elderly cohort, the generalizability of findings is limited. Future studies are needed assessing these relationships in a younger cohort, to evaluate if these findings are age-specific. Whilst this study presents novel relationships between the folate system, environmental UVR and skin pigmentation variants, these limitations should be examined in further studies to consider the health significance of these findings. 

## 5. Conclusions

This study examined independent and interactive influences of environmental UVR (4M-EDR) and pigmentation-related variants (*IRF4* rs12203592, *HERC2* rs12913832 and *MC1R* rs1805007) on folate and homocysteine levels. Environmental UVR levels and pigmentation-related variants *MC1R* rs1805007 and *IRF4* rs12203592 were shown to be independently associated with folate and homocysteine levels, however, no interactive influence between these factors was found. These findings highlight UVR exposure and skin pigmentation variants as emerging determinants of folate status in the Australian population. However, additional research is needed to assess if these findings are age- or population-dependent. Further, future research should consider the potential health significance of these findings, and assess influences of UVR and skin pigmentation-related variants on folate-related disease risks. 

## Figures and Tables

**Table 1:** PCR conditions for *HERC2* rs12913832 and *MC1R* rs1805007 variants.

Variant	Primers	Conditions	Enzyme	Expected Fragments
*HERC2* rs12913832	F:GAGGCCAGTTTCATTTGAGCTTTA R:CACCACTGGTAGTTTTCTTTGCC	95 °C 5min 35 cycles: 95 °C 30 s, 48 °C 30 s, 72 °C 30 s 72 °C 5 min	DraI	AA—203 bp AG—203, 226 bp GG—226 bp
*MC1R* rs1805007	F: CAAGAACCGGAACCTGCACT R: CCAGCATGTGGACGTACAGC	95 °C 5 min, 35 cycles: 95 °C 30 s, 48 °C 30 s, 72 °C 30 s 72 °C 5 min	HhaI	CC—197, 178, 42, 31 bp CT—197, 178, 74. 42, 31 bp

**Table 2:** Cohort characteristics.

	All (n = 599)	Male (n = 268)	Female (n = 331)
Age (years)^	77 (76–78)	77 (76–78)	77 (76–78)
RBC folate (nmol/L)^	1332.1 (1295.3–1368.9)	1312.8 (1259.9–1365.6)	1348.0 (1296.7–1399.2)
Serum folate (nmol/L)^	29.1 (28.2–30.0)	28.3 (27.0–29.6)	29.8 (28.5–31.1)
Homocysteine (μmol/L)^	10.4 (9.9–10.8)	10.8 (10.1–11.5)	10.0 (9.5–10.6)
Serum vitamin B12 (pmol/L)^	239.4 (227.2–251.6)	224.0 (208.7–239.2)^a^	252.0 (233.7–270.2)^a^
Creatinine (μmol/L)^	9.3 (8.9–9.7)	10.5 (9.8–11.1)^b^	8.4 (7.9–8.9)^b^
Folate intake (μg/day)^	736.4 (692.1–780.7)	727.6 (684.8–770.4)	743.6 (671.1–816.1)
Vitamin B_6_ intake (mg/d)^	9.4 (7.1–11.8)	10.1 (5.7–14.5)	8.9 (6.5–11.2)
Alcohol intake (g/day)^	8.6 (7.5–9.6)	13.3 (11.4–15.1)^c^	4.7 (3.9–5.6)^c^
Tea serves/day*			
<1	193 (32)	82 (31)	111 (34)
1–2	274 (46)	124 (46)	150 (45)
>2	132 (22)	62 (23)	70 (21)
Coffee serves/day*			
<1	208 (35)	91 (34)	117 (35)
1–2	262 (44)	120 (45)	142 (43)
>2	129 (22)	57 (21)	72 (22)
Smoking status*			
Never smoked	304 (51)	91 (34)^d^	213 (64)^d^
Current smoker	18 (3)	9 (3)	9 (3)
Ex-smoker	277 (46)	168 (63)^e^	109 (33)^e^
BMI category^			
Underweight	9 (2)	5 (2)	4 (1)
Normal	122 (22)	47 (19)	75 (25)
Overweight	237 (43)	119 (48)	118 (39)
Obese	185 (33)	78 (31)	107 (35)
4M-EDR#	16751.0(7788.2–29160.6)	16956.0(7788.2 -28258.0)	16585.07788.2–29160.6

Values within a row denoted with the same letter are significantly different (*p* < 0.05). ^ mean (95% CI); * n (%); #mean (range). RBC: red blood cell; BMI: body mass index, 4M-EDR; Erythemal dose rate accumulated over 4 months.

**Table 3:** Genotype frequencies for skin pigmentation variants in Retirement Health and Lifestyle Study (RHLS) cohort and comparison to 1000 Genomes European populations.

Variant	RHLS	EUR	GBR
	n (%)	n (%)	n (%)
*IRF4*-rs12203592			
CC genotype	359 (62)	395 (78)	61 (67)
CT genotype	188 (32)	99 (20)	27 (30)
TT genotype	33 (6)	9 (2)	3 (3)
*HERC2*-rs12913832			
AA genotype	24 (4)	90 (18)	4 (4)
AG genotype	178 (31)	186 (37)	25 (28)
GG genotype	364 (64)	227 (45)	62 (68)
*MC1R*-rs1805007			
CC genotype	473 (84)	435 (87)	75 (82)
CT genotype	87 (16)	64 (13)	14 (15)

EUR: European—1000 Genomes population, GBR: British in England and Scotland.

**Table 4:** Associations between 4 month accumulated erythemal dose rate (4M-EDR) and folate levels, with and without adjustments for folate determinants.

	RBC Folate Levels				Serum Folate Levels			
	*Unadjusted* *n* = *591*		Adjusted* n = *544*		*Unadjusted* *n* = *585*		Adjusted* n = *539*	
	p	β	p	β	p	β	p	β
4M-EDR	***<0.001***	***−0.19***	***<0.001***	***−0.19***	***0.045***	***−0.08***	***0.044***	***−0.08***

* Models adjusted for age, sex and known determinants of folate levels; dietary intake of folate and vitamin B_6_, alcohol, tea and coffee consumption, serum vitamin B_12_ levels, BMI and smoking status. Totals (*n*) vary due to missing data. Italic and bold highlight statistically significant results.

**Table 5:** Associations between 4M-EDR and homocysteine levels, with and without adjustments for homocysteine determinants.

	Homocysteine Levels			
	*Unadjusted* *n* = *570*		*Adjusted* *n* = *517*	
	p	β	p	β
4M-EDR	***<0.001***	***−0.28***	***<0.001***	***−0.28***

* Models adjusted for age, sex and known determinants of homocysteine levels; dietary intake of folate and vitamin B_6_, alcohol, tea and coffee consumption, serum vitamin B_12_ and creatinine levels, BMI and smoking status. Totals (*n*) vary due to missing data. Italic and bold highlight statistically significant results.

**Table 6:** Association between skin pigmentation variants and folate levels, with and without adjustments for folate determinants.

	RBC Folate Levels				Serum Folate Levels			
	*Unadjusted* *n* = *495*		Adjusted* n = *453*		*Unadjusted* *n* = *486*		Adjusted* n = *445*	
	p	β	p	β	p	β	p	β
*MC1R* rs1805007 (CC vs. CT)	0.429	−0.04	0.930	−0.00	***0.020***	−***0.11***	0.171	−0.06
*HERC2* rs12913832 (AG vs. GG)	0.919	0.00	0.466	−0.03	0.480	−0.03	0.130	−0.07
*IRF4* rs12203592 (CT vs. CC)	0.422	0.04	0.540	0.03	0.135	0.07	0.229	0.06

* Models adjusted for age, sex and known determinants of folate levels; dietary intake of folate and vitamin B_6_, alcohol, tea and coffee consumption, serum vitamin B_12_ levels, BMI and smoking status. Totals (*n*) vary due to missing data. Italic and bold highlight statistically significant results.

**Table 7:** Association between skin pigmentation genetic variants and homocysteine levels, with and without adjustments for homocysteine determinants.

	Homocysteine Levels			
	*Unadjusted* *n* = *460*		Adjusted* n = *436*	
	p	β	p	β
*MC1R* rs1805007 (CC vs CT)	0.715	0.02	0.725	0.02
*HERC2* rs12913832 (AG vs. GG)	0.208	−0.06	0.108	−0.08
*IRF4* rs12203592 (CT vs CC)	***0.026***	***−0.10***	0.059	−0.10

* Models adjusted for age, sex and known determinants of homocysteine levels; dietary intake of folate and vitamin B_6_, alcohol, tea and coffee consumption, serum vitamin B_12_ and creatinine levels, BMI and smoking status. Totals (*n*) vary due to missing data. Italic and bold highlight statistically significant results.

**Table 8:** Interactions between 4M-EDR and skin pigmentation variants in predicting folate levels.

	RBC Folate Levels				Serum Folate Levels			
	*Unadjusted* *n* = *495*		Adjusted* n = *453*		*Unadjusted* *n* = *486*		Adjusted* n = *445*	
	p	β	p	β	p	β	p	β
*4M-EDR*	***0.026***	***−0.15***	***0.034***	***−0.15***	0.156	−0.10	0.071	−0.13
*MC1R* rs1805007	0.460	−0.03	0.883	−0.01	***0.024***	***−0.10***	0.155	−0.07
*HERC2* rs12913832	0.788	−0.01	0.630	−0.02	0.434	−0.04	0.182	−0.06
*IRF4* rs12203592	0.286	0.05	0.420	0.04	0.112	0.07	0.214	0.06
*4M-EDR x MC1R* rs1805007	0.759	−0.02	0.669	−0.03	0.734	0.02	0.614	0.03
*4M-EDR x HERC2* rs12913832	0.594	0.03	0.504	0.03	0.721	−0.02	0.304	−0.05
*4M-EDR x IRF4* rs12203592	0.641	0.02	0.735	0.02	0.973	0.00	0.832	0.01

* Models adjusted for age, sex and known determinants of folate levels; dietary intake of folate and vitamin B_6_, alcohol, tea and coffee consumption, serum vitamin B_12_ levels, BMI and smoking status. Totals (*n*) vary due to missing data. Italic and bold highlight statistically significant results.

**Table 9:** Interactions between 4M-EDR and skin pigmentation variants in predicting homocysteine levels.

	Homocysteine Levels			
	*Unadjusted* *n* = *481*		Adjusted* *n* = *418*	
	p	β	p	β
*4M-EDR*	***<0.001***	***−0.30***	**<0.001**	**−0.30**
*MC1R*-rs1805007	0.728	0.02	0.896	0.01
*HERC2*-rs12913832	0.151	−0.06	0.171	−0.07
*IRF4*-rs12203592	0.073	−0.08	0.149	−0.07
*4M-EDR x MC1R* rs1805007	0.794	−0.02	0.646	−0.03
*4M-EDR x HERC2* rs12913832	0.804	0.01	0.879	0.01
*4M-EDR x IRF4* rs12203592	0.698	0.02	0.316	0.05

* Models adjusted for age, sex and known determinants of homocysteine levels; dietary intake of folate and vitamin B_6_, alcohol, tea and coffee consumption, serum vitamin B_12_ and creatinine levels, BMI and smoking status. Totals (*n*) vary due to missing data. Italic and bold highlight statistically significant results.
